# Characterization of Oxidative Modifications to Short Peptides Using Low Dose Rate X-Rays

**DOI:** 10.3390/app16062903

**Published:** 2026-03-18

**Authors:** Savannah Kidd, Thomas McCarthy, Simruthi Subramanian, Lieselotte Obst-Huebl, Jamie L. Inman, Sayan Gupta, Corie Y. Ralston

**Affiliations:** 1 Lawrence Berkeley National Laboratory, Molecular Foundry Division, Berkeley, CA 94720, USA; 2 Lawrence Berkeley National Laboratory, Accelerator Technology and Applied Physics Division, Berkeley, CA 94720, USA; 3 Lawrence Berkeley National Laboratory, Biological Systems and Engineering Division, Berkeley, CA 94720, USA; 4 Lawrence Berkeley National Laboratory, Molecular Biophysics and Integrated Bioimaging Division, Berkeley, CA 94720, USA

**Keywords:** hydroxyl radical footprinting, peptide oxidation, X-ray methods

## Abstract

The method of X-ray footprinting and mass spectrometry (XFMS) using high flux synchrotron X-ray sources has become an established method in structural biology and is based on the radiolytic production of hydroxyl radicals, which oxidatively modify protein sidechains. While other methods of producing hydroxyl radicals are available, one benefit of using high flux density sources is that hydroxyl radical scavenging reactions can be minimized, and exposure times kept short to minimize secondary reactions. Here we present an application of the XFMS method using low dose rate X-rays from a commercial instrument. We demonstrate the feasibility of the approach using short peptides, characterizing the oxidative modifications +14, +16, and +32 Da under both aerobic and low oxygen conditions, and we additionally quantify the hydrogen peroxide production for various doses using the low dose rate source. These results provide fundamental information on the oxidative damage to peptides due to hydroxyl radicals using a low dose rate X-ray source.

## 1. Introduction

With the structural biology method of X-ray footprinting mass spectrometry (XFMS), hydroxyl radicals (•OH) generated by the radiolysis of water produce oxidative modifications to biomolecules in solution. Peptide modifications are typically quantified using liquid chromatography mass spectrometry (LCMS), while further localization of the modifications to specific residues can be determined with MS2 analysis. With the XFMS method, the oxidation amounts are deliberately kept low so that the protein does not lose native conformation during exposure, and thus the map of modifications yields a solvent accessibility map. The solvent accessibility is in turn used to determine structural information, such as interaction regions between proteins and their binding partners, determination or validation of antibody–antigen binding sites, structural changes during protein complex formation, or changes in three-dimensional structure in folding or unfolding events [1].

Footprinting has also been explored with ionizing radiation sources other than X-rays, such as high-energy electrons [2] or gamma rays [3]. In addition, non-radiolytic methods of labeling proteins, such as using Fenton chemistry [4], are also common. Another high flux density method is the UV-laser-induced •OH labeling method known as Fast Photochemical Oxidation of Proteins (FPOP) [5,6], which relies on the dissociation of H_2_O_2_ to produce hydroxyl radicals. While there are many methods that rely on solvent accessibility as a means of determining structure, XFMS does not require the addition of extraneous reagents, and irradiation times can be on the order of microseconds. However, to the best of our knowledge, the XFMS method has not yet been explored using low dose rate X-rays. Low dose rate X-rays are readily available with commercial X-ray instruments, and so successful development of methods for conducting structural biology work using these instruments would be useful. In addition, low dose rate X-ray irradiation is currently the standard-of-care for cancer radiation treatment, and so information on oxidation of peptides and/or proteins under low dose rate irradiation is of interest to the radiation oncology field, especially in the context of the FLASH effect, as discussed later.

Fundamentally, the initial interaction of a photon with a water molecule is independent of dose rate: either an electron is ejected from a water molecule (photoelectric effect) or the water molecule goes into an excited state [7]. For the two national XFMS programs—one at the Advanced Light Source (ALS) at Lawrence Berkeley National Laboratory [8] and the other at the National Synchrotron Light Source II (NSLS-II) at Brookhaven National Laboratory [9]—the X-rays are high enough energy that the photoelectric effect dominates. After this initial event, the flux density and the linear energy transfer characteristics of the beam, as well as the concentration of dissolved O_2_ in solution, can affect the further evolution of radical species and their distribution in solution [10,11]. Therefore, we expect the subsequent radical–radical recombination to be different for low and high dose rate irradiation rates, and this in turn would affect the concentration of products such as solvated electrons, •OH, superoxide, and H_2_O_2_ during irradiation. Because these products react with protein side chains to varying degrees [12,13], we might expect to see differences in the amount and types of modification with low dose rate XFMS as compared to the traditional high dose rate XFMS method.

Several previous studies have tabulated modifications to short peptides or single amino acids using various radiation sources [14–16]. Here we report on the first use of low dose rate X-rays to generate oxidative modifications to short peptides under conditions used in the XFMS method, namely micromolar concentrations of peptide in phosphate buffer. We examine modification as a function of oxygen availability, and report on the generation of H_2_O_2_ in solution as a function of dose.

## 2. Materials and Methods

### 2.1. Peptide Preparation

Peptides were synthesized and purchased from GenScript in lyophilized form and guaranteed to ≥98% purity. They were dissolved in 1X phosphate buffer (VWR Chemicals, Radnor, PA, USA) to a concentration of 10 μM. Samples described as aerobic were prepared under ambient conditions. Samples described as low oxygen were prepared in a Coy Vinyl Anaerobic Airlock Chamber (Coy Laboratory Products Inc., Grass Lake, MI, USA), where each peptide solution, before aliquoting, was bubbled with N_2_ gas for three minutes to achieve an oxygen concentration of <0.1%, as measured by a FireSting-GO2 oxygen meter (PyroScience, Aachen, Germany). All samples were aliquoted into 0.25 mL PCR tubes containing 30 μL of peptide solution in each. Twenty-four samples per peptide were prepared (4 radiation doses in triplicate under aerobic and low oxygen conditions). Before transportation, while samples were still in the anaerobic chamber, samples were double-bagged in plastic bags with zip closures; they were first bagged by dose and then again by peptide, both for organizational purposes and to keep samples under low oxygen conditions for as long as possible. Aerobic samples were not bagged. All samples were transported on ice. A parallel test sample was prepared as described above, and then allowed to sit outside the anaerobic chamber for 15 min, corresponding to the maximum amount of time samples experienced being outside the plastic bags while being irradiated in the XRAD instrument. The test sample was then brought back into the anaerobic chamber and tested for oxygen, which measured at <0.5%.

### 2.2. Peptide Irradiation

The samples were irradiated at Lawrence Berkeley National Laboratory (LBNL) using the 320-kVp XRAD320 X-ray machine (Precision X-ray, Madison, CT, USA). The machine was operated at 300 kVp and 10 mA, with a 0.5 mm Cu beam hardening filter in place during irradiation. Samples were placed on a rotating platform in a circle to be equidistant from the center and irradiated with a dose rate of 0.020 Gy/s at varying times to achieve doses of 5, 10, and 15 Gy, with irradiation times of 4.2, 8.3, and 12.5 min, respectively. Dosimetry was performed with a NIST-traceable RadCal 10X6-0.18 ion chamber (~4% accuracy) calibrated to air kerma (RadCal, Monrovia, CA, USA) placed in the middle of the platform in XRAD cavity to measure the accumulated dose during the irradiation, as previously described [17]. Following irradiation, samples were immediately moved to a −80 °C freezer to minimize any ongoing modification resulting from secondary reactions and longer-lived radical species.

### 2.3. LC-MS/MS and Data Analysis

Peptides were analyzed using a Vanquish Flex UHPLC system coupled to an Orbitrap Exploris 480 mass spectrometer (ThermoFisher Scientific Inc., Waltham, MA, USA). Separation was performed on a Thermo Scientific Hypersil GOLD Peptide C18 column (2.1 × 100 mm, 1.9 μm particle size, maintained at 55 °C) at a flow rate of 0.400 mL/min with a mobile phase consisting of solvent A (0.1% formic acid in water) and solvent B (99.9% acetonitrile, 0.1% formic acid in water). The injection volume was 10 μL and peptides were eluted with the following gradient: the initial condition was 1% solvent B for a 3 min wash step with flow diverted to waste, after which solvent B was increased to 10% over 1.5 min, and then increased to 35% over 10 min followed by a ramp-up to 80% over 0.5 min, and held at 80% solvent B for 1.5 min at a flow rate of 0.600 mL/min. The flow rate was reduced to 0.400 mL/min at 2% solvent B for column equilibration. The mass spectrometer was operated in positive mode with full scan parameters set to an Orbitrap resolution of 60,000; mass range 300–1200 *m*/*z*; RF lens at 50%; AGC target at 3.0 × 10^6^; maximum injection time 60 ms. The top 10 most intense ions per MS scan were selected for HCD fragmentation with an intensity threshold of 5.0 × 10^3^ and dynamic exclusion set at 2 s. Data-dependent MS2 scan parameters included an isolation window of 2 *m*/*z*; 30% normalized collision energy; Orbitrap resolution set to 15,000; AGC target at 1.0 × 10^5^; maximum injection time 50 ms.

Peptide analysis was performed using the Byos^®^ software platform from Protein Metrics Inc. (Boston, MA, USA), which combines the Byonic^™^ MS/MS search engine and the Byologic^®^ peptide analysis software v5.8. The oxidative footprinting workflow has been customized to search for, identify, and quantify peptide modifications. Peptide modifications are reported as a percentage of modified peptide relative to unmodified (or native) peptide based on their respective extracted ion chromatogram (XIC) peak areas, and are calculated using the following formula:

$$\%\;\text{Modified}=\frac{{\text{XIC}\;\text{peak}\;\text{area}}_{\text{modified}}}{\left({\text{XIC}\;\text{peak}\;\text{area}}_{\text{modified}}+{\text{XIC}\;\text{peak}\;\text{area}}_{\text{native}}\right)}$$

This calculation is performed for each modification type and peptide. Once validated, peptide-level percent modified values were exported to Excel and shown as bar plots.

### 2.4. Hydrogen Peroxide Assay

AmplexRed (ThermoFisher Scientific Inc., Waltham, MA, USA) was used according to the manufacturer’s protocol for quantification of H_2_O_2_ in solution. Milli-Q water samples were prepared in triplicate. For low oxygen conditions, the samples were brought into a Coy Vinyl Anaerobic Airlock Chamber (Coy Laboratory Products Inc., Grass Lake, MI, USA) and N_2_ gas was bubbled through samples for 5 min, after which O_2_ levels were measured using a FireSting-GO2 oxygen meter (PyroScience, Aachen, Germany). All samples measured less than 1% O_2_ when bagged for transport to the XRAD instrument, as previously described [18]. After irradiation, 50 μL of each sample was pipetted into a 96-well plate, and 50 μL of the working buffer (AmplexRed solution) was added to each well. A BioTek Synergy H1 plate reader (Agilent, Santa Clara, CA, USA) was used to measure fluorescence, with excitation at 530 nm and emission measured at 590 nm. Hydrogen peroxide calibrants were prepared fresh for each experiment and used to generate a standard curve. We additionally measured the H_2_O_2_ concentration at several timepoints up to four hours after X-ray exposure, and found the concentration in solution was the same within error, showing that there was no longer production or reduction of H_2_O_2_ after X-ray exposure.

H_2_O_2_ was also used in concentrations ranging from 1 μM to 10 mM and incubated directly with the peptide for the amount of time corresponding to the maximum dose used in the peptide irradiation experiments. LCMS/MS was then conducted as described above.

## 3. Results

### 3.1. Measurement of Hydrogen Peroxide Due to Radiolysis

Radiolysis of aqueous solutions results in a complex reactive oxygen species (ROS) environment composed of many species, including electrons, hydroxyl radicals, superoxide, hydrogen ions, hydroperoxyl radicals, and hydrogen peroxide [19,20] (Table S1). Simulations have been used to predict the evolution and lifetime of various species under different dose rates and radiation types, and experimental methods to quantify yields of specific species have included fluorescence readouts, EPR spin trapping, and scavenging reactions [10,21,22]. Hydrogen peroxide, although one of the least reactive species to proteins, is stable and long-lasting, can be measured after irradiation using the standard fluorescence assay AmplexRed (ThermoFisher Scientific Inc., Waltham, MA, USA). Using this assay, previous studies using electron, carbon ion, and proton sources have shown that under irradiation, samples low in dissolved oxygen will produce less H_2_O_2_ than fully aerobic samples [10,23–26]. However, results are complicated by the fact that the dose rate and linear energy transfer (LET) value of the radiation will affect the production and evolution of radiolytic species. The presence of radical scavengers and dissolved gases such as CO_2_ will also affect the final yield of H_2_O_2_ [23], and simulations have produced conflicting results [27–30].

**Figure 1.**
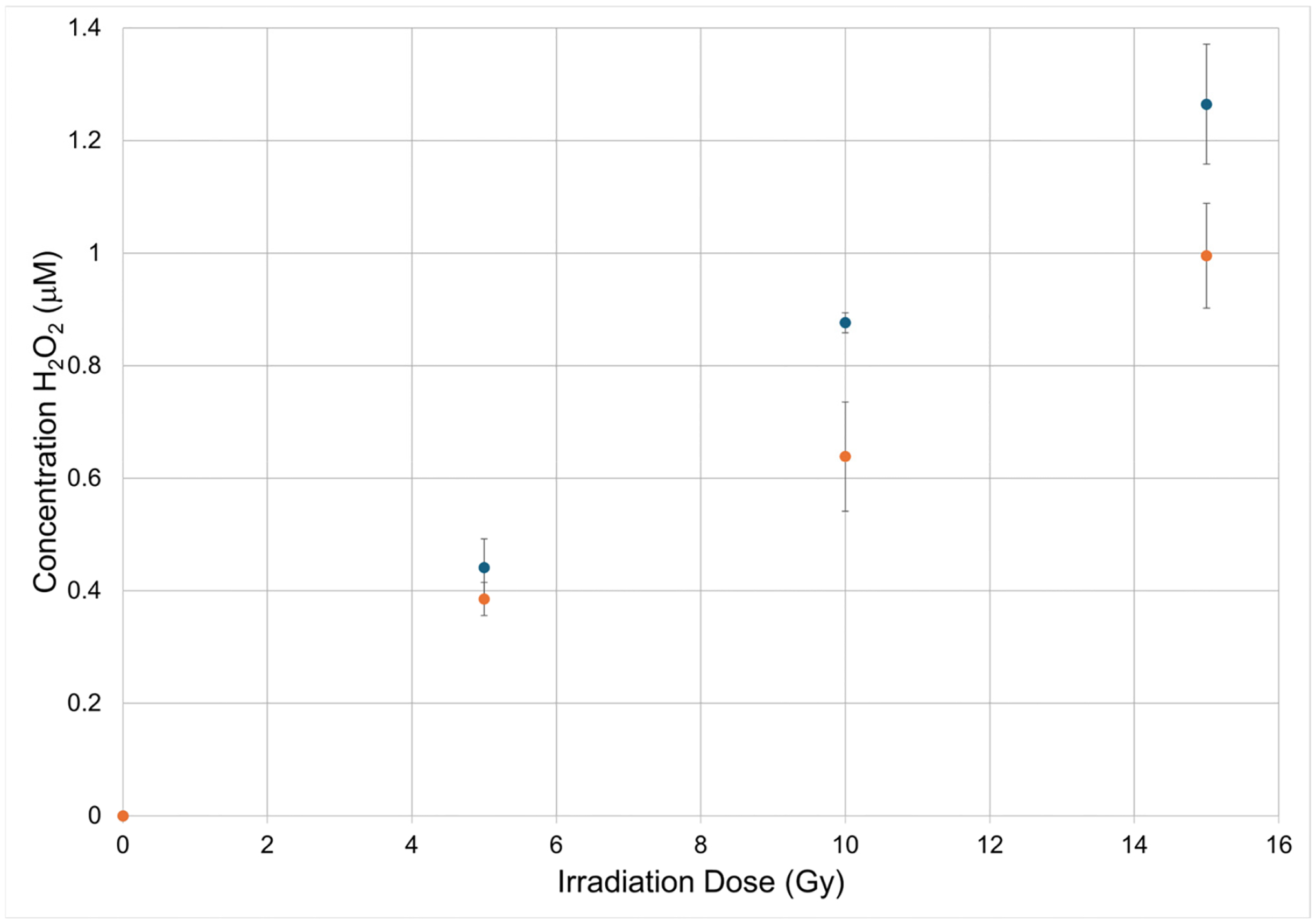
Hydrogen peroxide production in aerobic (blue) or low oxygen (orange) Milli-Q water as a function of dose using 0.02 Gy/s dose rate X-rays. Error bars represent the standard deviation between triplicate data points.

Because of the long exposure times necessary when using a low dose rate source, and the corresponding potential buildup of H_2_O_2_, we characterized the H_2_O_2_ yield for the exposures used in this study under both aerobic and low oxygen conditions. For a given dose, aerobic milli-Q produced higher yields of H_2_O_2_ than low oxygen samples under X-ray irradiation, with higher doses leading to a larger difference in final yield (Figure 1). We also compared milli-Q water with 10 mM Tris buffer and 10 mM Phosphate buffer and observed no differences in H_2_O_2_ yield (Table S2). To further test the effect of the ROS environment on protein side chains, we then designed short peptides and characterized their oxidation under X-ray irradiation with different doses and two different dissolved oxygen conditions as described below.

### 3.2. Peptide Design

We designed short peptides for this study in order to minimize secondary structure. Single amino acids have varying reactivities to hydroxyl radicals, with the highest reactivity observed for the sulfur-containing residues and the aromatic residues. We therefore kept the sequence of the peptides identical except for one residue, which varied an order of magnitude in reactivity. We also included specific residues to maintain solubility of the peptide, and hydrophobic residues to ensure good LC coverage. The specific sequence was EDLAXLK, in which X represents residues F, G, H, I, M, P, R, or Y.

To test whether H_2_O_2_ contributed to oxidative modification, we subjected the most reactive peptide (EDLAMLK, or peptM) to H_2_O_2_ in a concentration similar to that determined using the AmplexRed assay as well as higher concentrations of H_2_O_2_. At 1 μM concentration, we observed no increase in oxidation on peptM over background oxidation, and only at concentrations of 1 mM and higher did we observe any significant oxidation over background (Table S3). While Met can be oxidized directly by H_2_O_2_, the reaction rate is ~10^*−*2^ M^*−*1^s^*−*1^ [13] which is 7–8 orders of magnitude lower than the reaction rate with hydroxyl radical [7].

Another mechanism by which oxidation of peptides and proteins in solution might occur is through hydroxyl radicals generated by reaction of H_2_O_2_ with trace metals in solution [31]. However, because we did not observe oxidation of peptM in solution containing H_2_O_2_ at the concentrations generated during X-ray irradiation, we conclude that oxidation of the peptides in this study was primarily due to attack by hydroxyl radicals. This is also the assumption in typical XFMS experiments using high dose rate X-rays [8].

### 3.3. Peptide Oxidation Under Aerobic Conditions

**Figure 2.**
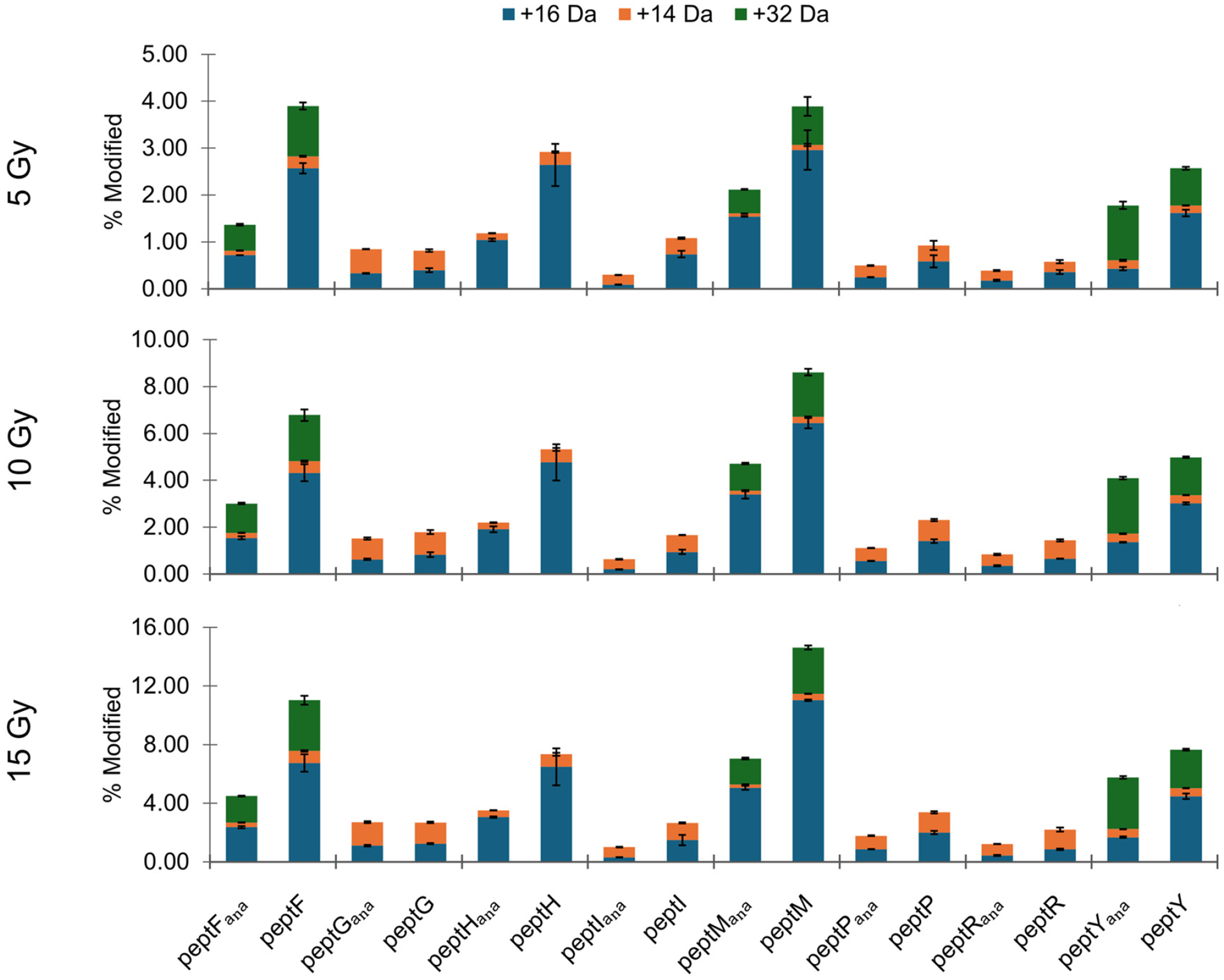
Peptide-level percent modified by modification type and radiation dose. Organized by peptide, where peptX (X representing the fifth position residue which distinguishes each peptide) refers to aerobically prepared samples, and peptX_ana_ refers to low oxygen prepared samples. For each peptide, from left to right, percent modified is shown for each dose: 5, 10, and 15 Gy. As this data has been averaged and normalized to 0% modification at 0 Gy, this dose is not shown on this plot. Error bars represent the standard deviation between triplicate data points.

Figure 2 and Table S4 show percent modification for each peptide as a function of dose (5, 10 and 15 Gy), oxygen availability, and type of oxidative modification (+14, +16, and +32 Da). Representative XICs and fragment ion spectra are shown in Figures S1–S11. The +16 Da modification is a hydroxylation and is the most common modification reported in XFMS and FPOP studies [1]. However, reaction mechanisms for the various types of modifications are distinct for each amino acid and can include the +14, +32 modifications in addition to higher mass additions and some subtractions. The +14 Da oxidation, referred to as a carbonyl modification, results from formation of a C=O bond after hydroxyl radical abstraction of a H from a saturated carbon, and subsequent attack by O_2_. The +32 Da modification in this study refers to a double hydroxyl addition at two different carbons within a single residue, or in the case of Met, to a double oxidation of the sulfur resulting in a sulfone.

In this study, modification of a given type (+14, +16, or +32 Da) ranged from 0.05–11%, with modification increasing linearly with dose. For high dose rate XFMS, exposures are also chosen to produce a linear dose response, and are kept deliberately low to reduce unfolding of the protein due to oxidation [8]. Here, the highest amount of oxidation (5–11%) was observed for the +16 Da oxidation on the peptides containing Met or an aromatic ring (the peptF, peptH, and peptY peptides). For these peptides, the +16 Da modification was on the order of 10-fold higher than the +14 Da modification and approximately double that of the +32 Da modification. For all the peptides characterized in this study, the +14 Da modifications ranged from 0.5 to 1.5% for the highest X-ray dose. We note also that the +14 Da product can result from a +32 Da addition and subsequent loss of water. Of the peptides that did not contain S or an aromatic ring, the hydroxylation modification was significantly lower (0.8–1.5% at the highest X-ray dose), and in the case of the peptG and peptR peptides, on the order of or even lower than the carbonyl modification. Previous studies have shown that depending on sequence, the oxidation of short peptides can result in higher carbonyl products than hydroxyl products [14]. We also found that the amount of hydroxylation of peptF is higher than hydroxylation of peptY (6.7 vs. 4.5%). While Phe has been reported to have a lower reactivity to hydroxyl radicals than Tyr when in the free amino acid form [7], other studies have shown that the yield of hydroxylated Phe due to hydroxyl radical attack can increase when in a short peptide [14].

### 3.4. Peptide Oxidation Under Low Oxygen Conditions

When prepared and irradiated under low oxygen, the peptides generally exhibited lower oxidation at each dose relative to the aerobically prepared peptides, as has been previously observed in high dose rate XFMS [1]. The +16 Da oxidation ranged from 0.3 to 5%, with the highest yields observed on peptM and the peptides containing an aromatic ring (Table S4), similar to the trend for the aerobic peptides.

**Figure 3.**
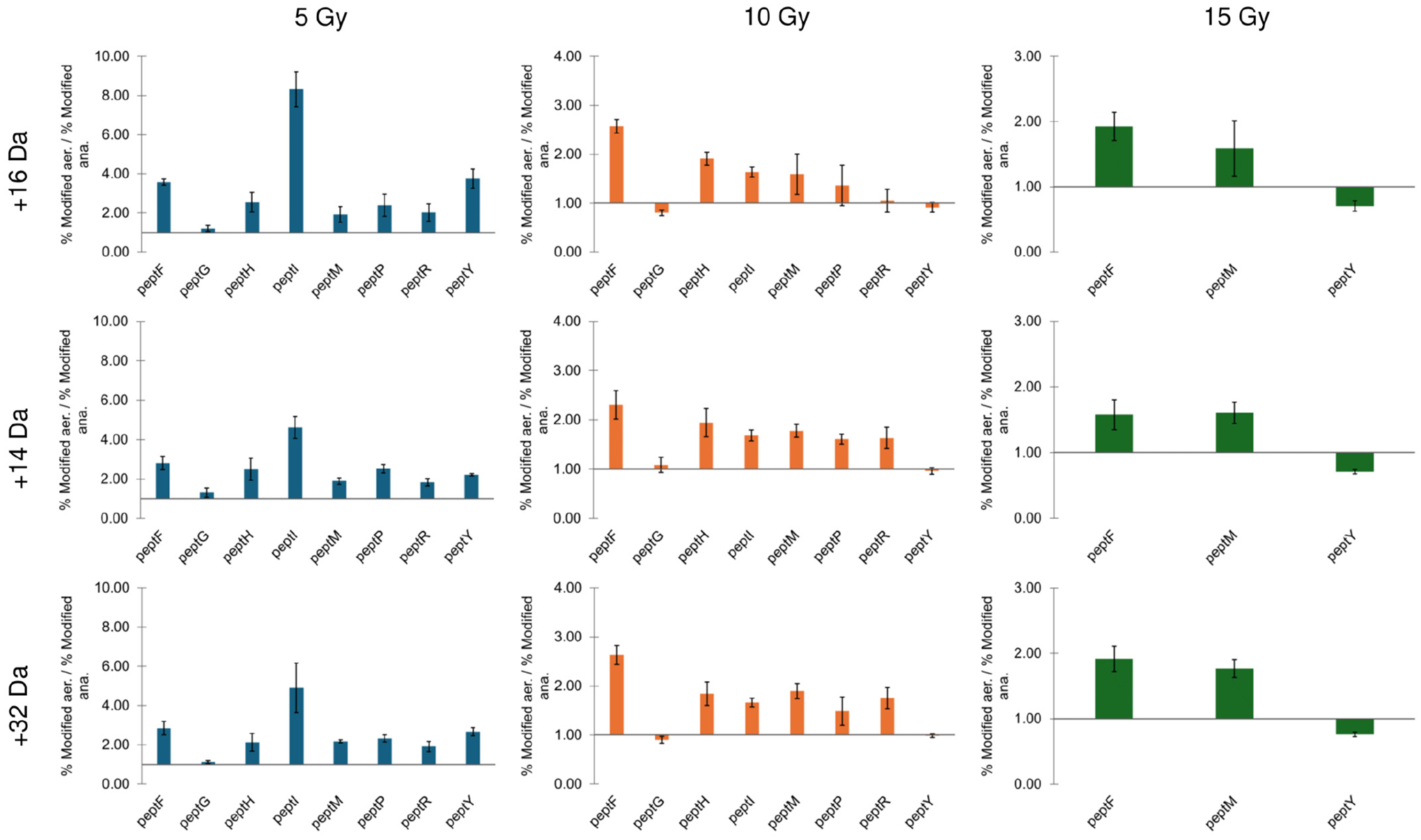
Ratios of peptide-level percent of modification under aerobic to low oxygen conditions, separated by type of modification and dose. The blue bars (**top row**) show only +16 Da, the orange bars (**middle row**) show only +14 Da, and the green bars (**bottom row**) show only the +32 Da modification. From left to right, columns are 5, 10, and 15 Gy exposures. Error bars represent the standard deviation between triplicate data points.

The calculated ratios of percent oxidation for aerobic versus low-oxygen preparation for each peptide in this study are shown in Figure 3, separated by modification type and dose. The general pattern of oxidation does not change with the dose (5, 10, or 15 Gy). For most of the peptides and modification type, ratios were above one, indicating more oxidation under aerobic conditions, with some exceptions. One notable exception is peptY, which showed more +32 Da oxidation under low oxygen conditions. The yield of the first hydroxylation of peptY is comparable to the double hydroxylation of peptF: For peptY, 4.5% (aerobic) to 1.7% (low oxygen), and for peptF, 3.8% (aerobic) to 1.8% (low oxygen). However, the second hydroxylation of peptY is 2.7% (aerobic) to 3.5% (low oxygen). The reaction scheme for hydroxylation of an aromatic ring has been delineated in several sources [7] and begins with a direct attack by a hydroxyl radical on the aromatic ring. The resulting carbon-centered radical is in turn susceptible to attack by O_2_, which is followed by hydroperoxide elimination, leaving the hydroxylated form of the amino acid. Previous studies on free amino acids or three-residue peptides have found that in the absence of O_2_, the final hydroxylated product is still formed, but in lower yield, presumably because the hydroxylated radical intermediate is not stable [7]. A previous study using O^18^ labelling showed that oxidation of Phe and Tyr could occur through multiple mechanisms, not all involving oxygen [32]. We have seen in a previous study [18] that some Phe residues in a protein show increased hydroxylation under low oxygen conditions, while others do not, indicating that the local environment plays a significant role in hydroxylation of an aromatic ring. It is possible that for the peptides used in this study, the neighboring residues play a role in stabilizing the initial OH addition to the ring, mediating the dependency on O_2_.

## 4. Discussion

Free amino acids have been demonstrated to react at different rates to different hydroxyl radicals, H_2_O_2_ and electrons with the reactivities of the amino acids specifically to hydroxyl radicals ranked as follows. For the residues used in this particular study, the order is as follows: Tyr > Met > Phe > His > Arg > Ile > Leu > Pro > Lys > Glu > Ala > Asp > Gly [7]. Since the peptides used in this study differed only in their fifth residue, we assume that the change in peptide-level modification between the eight peptide sequences is due to the amino acid occupying the fifth position on the peptide. If we rank the peptides based on total peptide-level percent modified observed, then we list the peptides as follows: peptM > peptF > peptY > peptH > peptP > peptG > peptI > peptR. That this does not completely align with the ranking of reported reactivities of individual amino acids to hydroxyl radicals is perhaps not surprising, given that the local sequence of a residue can alter peptide reactivity/modification, as has been noted in some previous studies [14,15].

Here we have shown that peptide modification under low dose rate X-ray irradiation using a commercial instrument (0.02 Gy/s) results in modification types of +14, +16, +32 Da, and amounts of up to 11% at a given dose. These are sufficient for XFMS analysis; however, it should be noted that the low dose rate source required much longer irradiation times than at high dose rate beamlines (minutes versus milliseconds) to achieve the same dose. For proteins under long irradiation times, we might expect to see secondary oxidation reactions accumulating, which would limit the structural information obtainable using a low dose rate source. Therefore, low dose rate X-rays may not be a viable alternative approach for XFMS, which is based on high dose rate synchrotron X-ray sources. To ascertain whether low dose rate XFMS could be applied to obtain structural information on proteins, one would have to first determine whether enough modification could be obtained in a reasonable amount of time, since the higher the protein content in solution, the lower the amount of oxidative damage for a given dose. Second, one would have to conduct additional biophysical measurements after X-ray exposure to determine whether the proteins unfolded during the experiment. Such studies would determine whether XFMS was possible with a low dose rate instrument, and ideally would also investigate the range of protein sizes it would be possible to interrogate using the method.

The measurement of oxidation to peptides under low dose rate X-rays is also of interest to the radiation oncology field, since all X-ray instruments currently used for cancer treatment are low dose rate sources. The use of high dose rate sources for cancer treatment has been the subject of intense recent research because of the so-called “FLASH” effect, in which high dose rate radiation will spare healthy tissue to a greater extent than equivalent doses at the conventional low dose rates, while still maintaining tumor control [33]. The FLASH effect has been demonstrated using various radiation sources including electron, proton, and heavy ion radiation, and X-rays [34]. Future studies comparing oxidation of peptides and proteins under low dose rate X-ray irradiation versus high dose rate X-ray irradiation will therefore be of interest to the radiation oncology field, and this study lays the groundwork for future work in this area.

We also measured the concentration of hydrogen peroxide resulting from low dose rate exposures in order to rule out the possibility that prolonged exposure to hydrogen peroxide formed during exposure might contribute to oxidation products. We found that for the highest dose (15 Gy), the H_2_O_2_ produced was 1.0–1.3 μM, depending on oxygen availability. Exposing the most reactive peptide, peptM, to up to 100 μM concentration of H_2_O_2_ for the longest exposure time (15 min) did not result in oxidation over background. In addition, we found that the production of H_2_O_2_ was similar in milli-Q water, Phosphate buffer, and Tris buffer. One of the mechanisms for production of H_2_O_2_ is the combination of two hydroxyl radicals, so it is noteworthy that the H_2_O_2_ yield in Tris, a known hydroxyl radical scavenger, was comparable to the yield in pure water or Phosphate buffer. This points to other H_2_O_2_-producing mechanisms possibly playing a greater role, such as the combination of hydroperoxyl radicals or ions with protons or hydrogen ions, all of which are produced under radiolysis of aqueous solutions (Table S1). However, the production of hydroperoxyls depends to some extent on the presence of O_2_, which may explain the lower H_2_O_2_ yield in solutions with low dissolved O_2_. Molecular oxygen also serves as an electron scavenger, and since electrons react with hydroxyl radicals to produce hydroxyl ions, reducing the available electrons in solution can increase the amount of hydroxyl radicals, possibly increasing in turn the yield of H_2_O_2_. These considerations are relevant for structural biology studies using the method of hydroxyl radical footprinting, since this method relies on hydroxyl radical-induced modifications of protein sidechains, and is based on the assumption that protein modifications are not affected by H_2_O_2_ generated during radiolysis. The measurement of H_2_O_2_ yield under different oxygen levels is also relevant for radiation oncology, since the tumor environment is generally more hypoxic than healthy tissue, and H_2_O_2_ is one of the oxidatively damaging species produced during radiation treatments.

## Supplementary Material

Supplementary Information

The following supporting information can be downloaded at: https://www.mdpi.com/article/10.3390/app16062903/s1, Table S1: Radiolysis reactions and rate constants in aqueous buffer; Table S2: Production of hydrogen peroxide in water, Phosphate, and Tris buffers; Table S3: Percentage oxidation of EDLAMLK peptide under exposure to hydrogen peroxide; Table S4: Percent of modified peptide by modification type, radiation dose and oxygen availability. Figure S1: Extracted ion chromatograms (XICs) and representative MS/MS plots for peptG. Figure S2: Extracted ion chromatograms (XICs) and representative MS/MS plots for peptH. Figure S3: Extracted ion chromatograms (XICs) and representative MS/MS plots for peptI. Figure S4: Extracted ion chromatograms (XICs) and representative MS/MS plots for peptP. Figure S5: Extracted ion chromatograms (XICs) and representative MS/MS plots for peptR. Figure S6: Extracted ion chromatograms (XICs) and representative MS/MS plots for aerobically prepared peptF. Figure S7: Extracted ion chromatograms (XICs) and representative MS/MS plots for low oxygen prepared peptF. Figure S8: Extracted ion chromatograms (XICs) and representative MS/MS plots for aerobically prepared peptM. Figure S9: Extracted ion chromatograms (XICs) and representative MS/MS plots for low oxygen prepared peptM. Figure S10: Extracted ion chromatograms (XICs) and representative MS/MS plots for aerobically prepared peptY. Figure S11: Extracted ion chromatograms (XICs) and representative MS/MS plots for low oxygen prepared peptY. References [19,35,36] are cited in the supplementary materials.

## Data Availability

All data are available from the corresponding author upon reasonable request.

## Abbreviations

The following abbreviations are used in this manuscript:

LCMS: Liquid Chromatography Mass Spectrometry
XFMS: X-ray Footprinting Mass Spectrometry
