# Supplementary material for: Characterization of Oxidative Modifications to Short Peptides Using Low Dose Rate X-Rays
Source: Appl Sci (Basel). Author manuscript; Available in PMC 2026 Jul 8. (PMC13340869; doi:10.3390/app16062903)
Supplement: Supplementary Information [file NIHMS2191440-supplement-Supplementary_Information.pdf]

Table S1: Radiolysis reactions and rate constants in aqueous buffer. Listed are reactions with rates 1.00E+07 and above. Reactions either producing or consuming H_2O_2 are highlighted.

Reactant 1	Reactant 2	Product 1	Product 2	Product 3	Rate (mol^{-1} s^{-1})
e_{aq}	e_{aq}	H_2	$2OH^-$		5.50E+09 (a)<br>6.36E+09 (b)<br>5.00E+09 (c)
e_{aq}	$H\cdot$	H_2	OH^-		2.50E+10 (a, b, c)
e_{aq}	H^+	$H\cdot$			2.30E+10 (a)<br>2.11E+10 (b)<br>2.20E+10 (c)
e_{aq}	$HO_2\cdot$	HO_2^-			1.29E+10 (b)
e_{aq}	HO_2^-	$O^{\cdot-}$	OH^-		3.50E+09 (b, c)
e_{aq}	H_2O_2	$OH\cdot$	OH^-		1.10E+10 (a, b)<br>1.20E+10 (c)
e_{aq}	$O^{\cdot-}$	$2OH^-$			2.20E+10 (a)<br>2.31E+10 (b)
e_{aq}	O_2	$O_2^{\cdot-}$			1.90E+10 (a, c)<br>1.74E+10 (b)
e_{aq}	$O_2^{\cdot-}$	O_2^{2-}			1.30E+10 (a)
e_{aq}	$O_2^{\cdot-}$	H_2O_2	$2OH^-$		1.29E+10 (b)
e_{aq}	$O_2^{\cdot-}$	HO_2^-	OH^-		1.30E+10 (c)
e_{aq}	$OH\cdot$	OH^-			3.00E+10 (a, b, c)
$H\cdot$	$H\cdot$	H_2			7.75E+09 (a)<br>5.03E+09 (b)<br>1.00E+10 (c)
$H\cdot$	H_2O_2	H_2O	$OH\cdot$		9.00E+07 (a, c)<br>3.50E+07 (b)
$H\cdot$	$HO_2\cdot$	H_2O_2			1.00E+10 (a, b)<br>2.00E+10 (c)
$H\cdot$	O_2	$HO_2\cdot$			2.10E+10 (a, b)<br>1.80E+10 (c)
$H\cdot$	$O^{\cdot-}$	OH^-			2.00E+10 (b)
$H\cdot$	$O_2^{\cdot-}$	HO_2^-			1.00E+10 (b)<br>2.00E+10 (c)
$H\cdot$	OH^-	e_{aq}	H_2O		2.20E+07 (a)<br>2.51E+07 (b)<br>2.10E+07 (c)
$H\cdot$	$OH\cdot$	H_2O			7.00E+09 (a)<br>1.55E+10 (b)<br>2.00E+10 (c)
H^+	HO_2^-	H_2O_2			5.00E+10 (b)<br>2.00E+10 (c)
H^+	$O^{\cdot-}$	$OH\cdot$			4.78E+10 (b)
H^+	OH^-	H_2O			1.13E+11 (b)<br>1.43E+11 (c)

H^+	$O_2^{\cdot-}$	$HO_2^{\cdot}$			4.78E+10 (b)<br>4.50E+10 (c)
$HO_2^{\cdot}$	$O_2^{\cdot-}$	O_2	HO_2^-		9.70E+07 (b)<br>8.90E+07 (c)
$HO_2^{\cdot}$	OH^-	$O_2^{\cdot-}$	H_2O		6.30E+09 (b)
$HO_2^{\cdot}$	$OH^{\cdot}$	H_2O	O_2		6.00E+09 (a)<br>7.90E+09 (b)<br>1.20E+10 (c)
HO_2^-	$OH^{\cdot}$	$HO_2^{\cdot}$	OH^-		7.50E+09 (a)<br>8.32E+09 (b)<br>5.00E+09 (c)
HO_2^-	$O^{\cdot-}$	$O_2^{\cdot-}$	OH^-		4.00E+08 (a)<br>3.50E+08 (b)<br>8.00E+08 (c)
$O^{\cdot-}$	H_2	$H^{\cdot}$	OH^-		8.00E+07 (a, c)<br>1.21E+08 (b)
$O^{\cdot-}$	H_2O_2	H_2O	$O_2^{\cdot-}$		5.00E+08 (a)<br>2.00E+08 (c)
$O^{\cdot-}$	$O_2^{\cdot-}$	O_2	$2OH^-$		6.00E+08 (a, b)
$O^{\cdot-}$	O_2	O_3^-			3.60E+09 (a)<br>3.00E+09 (c)
$O_3^{\cdot-}$	$O^{\cdot-}$	$2O_2^{\cdot-}$			7.00E+08 (b)
$O_3^{\cdot-}$	H^+	$OH^{\cdot}$	O_2		9.00E+10 (b)
$OH^{\cdot}$	H_2O_2	$HO_2^{\cdot}$	H_2O		2.70E+07 (a)<br>3.30E+07 (c)
$OH^{\cdot}$	H_2	$H^{\cdot}$	H_2O		4.20E+07 (a)<br>3.28E+07 (b)<br>3.60E+07 (c)
$OH^{\cdot}$	$O_2^{\cdot-}$	O_2	OH^-		8.00E+09 (a)<br>1.07E+10 (b)<br>9.00E+09 (c)
$OH^{\cdot}$	$OH^{\cdot}$	H_2O_2			5.50E+09 (a, b, c)
$OH^{\cdot}$	OH^-	$O^{\cdot-}$	H_2O		1.30E+10 (a)<br>6.30E+09 (b)<br>1.20E+10 (c)
$OH^{\cdot}$	$O^{\cdot-}$	HO_2^-			2.00E+10 (a)<br>1.00E+09 (b)
$OH^{\cdot}$	$O_3^{\cdot-}$	$O_2^{\cdot-}$	$HO_2^{\cdot}$		8.50E+09 (b)
$OH^{\cdot}$	$H_2O_2^+$	H_3O^+	O_2		1.20E+10 (a)
OH^-	H_2O_2	HO_2^-	H_2O		4.71E+08 (b)

a = Buxton, Greenstock, Helman, Ross, Journal of Physical and Chemical Reference Data 17, 513, 1988.
b = Ianik Plante, Phys Med Biol 66 03TR02, 2021
c = Boyd, Carver, Dixon, Radiat Phys Chem Vol 15, pp177-185, 1980.

Table S2: Production of hydrogen peroxide in milli-Q water, Phosphate, and Tris buffer.

Dose (Gy)	[H_2O_2], mM; Average of 3 measurements in aerobic water	[H_2O_2], mM; Average of 3 measurements in low oxygen water	[H_2O_2], mM; Average of 3 measurements in aerobic 10 mM Phosphate buffer, pH 7.4	[H_2O_2], mM; Average of 3 measurements in aerobic 10 mM Tris buffer, pH 7.4
5	0.44 ± 0.05	0.39 ± 0.03	0.40 ± 0.06	0.47 ± 0.10
10	0.88 ± 0.02	0.64 ± 0.10	0.85 ± 0.02	1.19 ± 0.34
15	1.26 ± 0.11	0.99 ± 0.09	1.29 ± 0.14	1.47 ± 0.17

Table S3: Percentage oxidation of EDLAMLK peptide under exposure to hydrogen peroxide.

[H_2O_2], mM	Percent +16Da modification; Average of 3 measurements	Std deviation
0	1.99	0.04
0.01	1.85	0.15
0.1	2.14	0.06
1	3.60	0.19
10	15.93	1.37
100	72.47	6.07

Table S4: Percent of modified peptide by modification type, radiation dose and oxygen availability. Data in the table has been averaged for each dose and normalized to 0% modified peptide at 0 Gy. Error shown is the standard deviation of three replicates per dose. Except for where an error value is given, the error is <0.001. R is the ratio of aerobic to low oxygen percent modification for 15 Gy for each modification type.

	+16 Da					+14 Da					+32 Da				
	0 Gy*	5 Gy	10 Gy	15 Gy	R	0 Gy*	5 Gy	10 Gy	15 Gy	R	0 Gy*	5 Gy	10 Gy	15 Gy	R
peptF	0 ± 0.0016	2.57 ± 0.11	4.32 ± 0.36	6.74 ± 0.59	2.84 ± 0.34	0	0.252 ± 0.011	0.495 ± 0.036	0.834 ± 0.051	2.63 ± 0.19	0	1.18 ± 0.09	2.16 ± 0.27	3.77 ± 0.34	2.09 ± 0.21
peptF$_{ana}$	0 ± 0.0248	0.715 ± 0	1.54 ± 0.06	2.37 ± 0.08		0	0.0984 ± 0.0012	0.215 ± 0.011	0.317 ± 0.004		0	0.556 ± 0.019	1.25 ± 0.03	1.80 ± 0.02	
peptG	0	0.397 ± 0.044	0.823 ± 0.104	1.24 ± 0.03	1.12 ± 0.07	0	0.415 ± 0.030	0.967 ± 0.084	1.45 ± 0.05	0.90 ± 0.06					
peptG$_{ana}$	0	0.329 ± 0.006	0.629 ± 0.033	1.11 ± 0.04		0	0.517 ± 0.002	0.889 ± 0.050	1.61 ± 0.06						
peptH	0	2.64 ± 0.45	4.76 ± 0.77	6.48 ± 1.25	2.12 ± 0.45	0	0.275 ± 0.015	0.554 ± 0.061	0.856 ± 0.093	1.84 ± 0.24					
peptH$_{ana}$	0	1.04 ± 0.03	1.91 ± 0.12	3.05 ± 0.06		0	0.144 ± 0.002	0.285 ± 0.011	0.465 ± 0.010						
peptI	0 ± 0.0023	0.738 ± 0.072	0.943 ± 0.092	1.50 ± 0.35	4.90 ± 1.26	0 ± 0.0011	0.340 ± 0.016	0.722 ± 0.015	1.16 ± 0.03	1.65 ± 0.08					
peptI$_{ana}$	0	0.0887 ± 0.0009	0.205 ± 0.004	0.306 ± 0.007		0	0.208 ± 0.003	0.429 ± 0.019	0.701 ± 0.017						
peptM	0 ± 0.1121	2.96 ± 0.42	6.44 ± 0.22	11.0 ± 0.1	2.18 ± 0.08	0	0.108 ± 0.026	0.270 ± 0.011	0.434 ± 0.017	1.90 ± 0.16	0	0.825 ± 0.200	1.90 ± 0.14	3.16 ± 0.14	1.80 ± 0.14
peptM$_{ana}$	0 ± 0.0963	1.54 ± 0.01	3.40 ± 0.18	5.05 ± 0.14		0	0.0678 ± 0.0013	0.152 ± 0.005	0.229 ± 0.010		0	0.509 ± 0.009	1.16 ± 0.03	1.76 ± 0.06	
peptP	0	0.584 ± 0.131	1.40 ± 0.09	2.00 ± 0.12	2.33 ± 0.17	0	0.341 ± 0.097	0.903 ± 0.044	1.38 ± 0.07	1.49 ± 0.11					
peptP$_{ana}$	0	0.244 ± 0.003	0.556 ± 0.012	0.857 ± 0.012		0	0.250 ± 0.005	0.560 ± 0.008	0.925 ± 0.021						

peptR	0	0.357 ± 0.046	0.650 ± 0.010	0.852 ± 0.043	1.19 ± 0.26	0	0.221 ± 0.037	0.789 ± 0.049	1.35 ± 0.14	1.76 ± 0.21					
peptR$_{ana}$	0	0.177 ± 0.016	0.355 ± 0.030	0.445 ± 0.038		0 ± 0.0012	0.211 ± 0.012	0.484 ± 0.034	0.769 ± 0.014						
peptY	0 ± 0.0021	1.61 ± 0.07	3.01 ± 0.05	4.48 ± 0.19	2.67 ± 0.19	0 ± 0.0028	0.162 ± 0.003	0.350 ± 0.007	0.552 ± 0.009	0.98 ± 0.04	0	0.816 ± 0.033	1.66 ± 0.03	2.67 ± 0.06	0.76 ± 0.04
peptY$_{ana}$	0 ± 0.0013	0.430 ± 0.037	1.36 ± 0.01	1.68 ± 0.05		0 ± 0.0034	0.178 ± 0.016	0.364 ± 0.018	0.561 ± 0.013		0	1.16 ± 0.08	2.35 ± 0.06	3.51 ± 0.09	

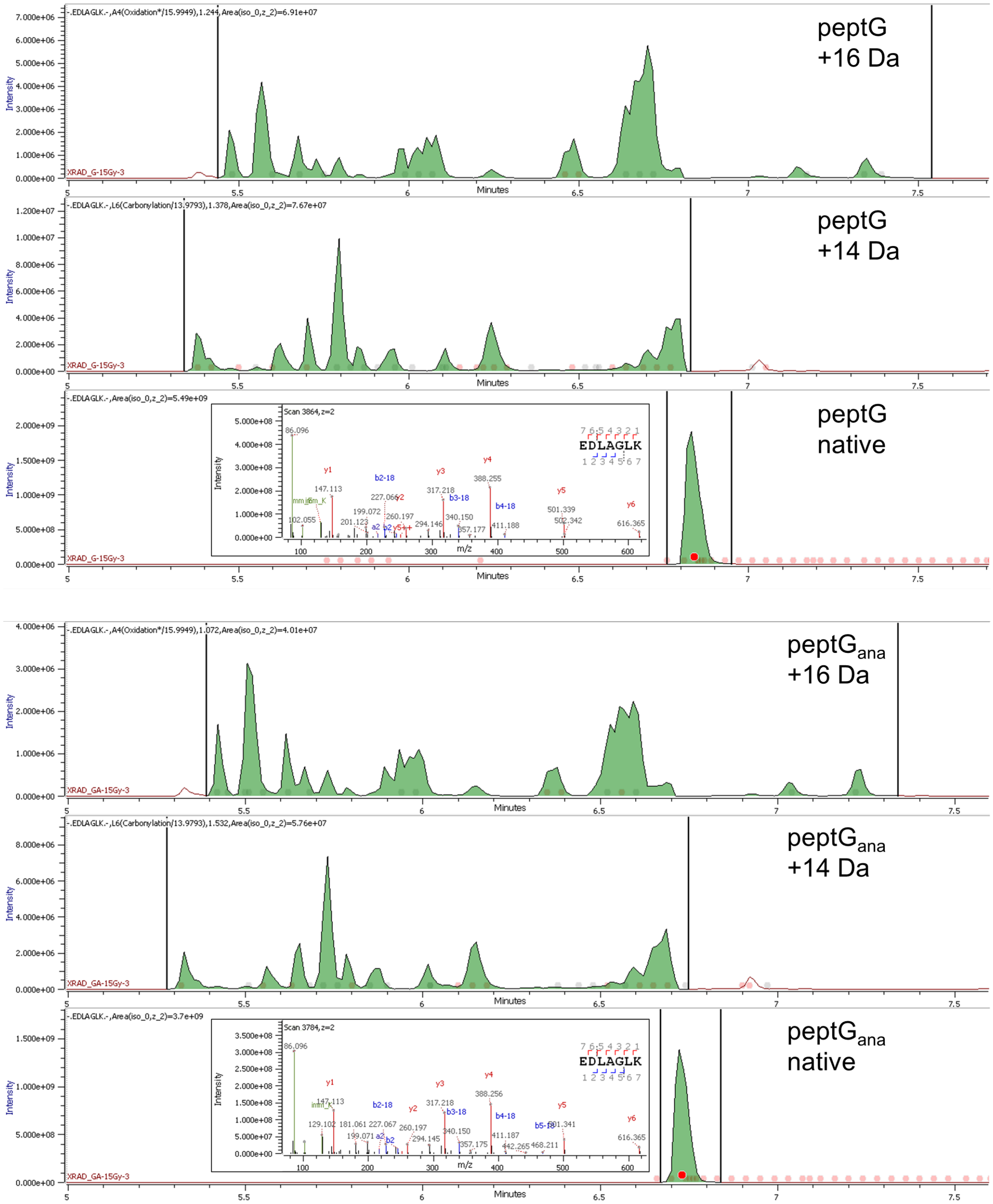

Figure S1: Extracted ion chromatograms (XICs) and representative MS/MS plots for peptG. From top to bottom: XICs for aerobically prepared peptide (+16Da, +14Da, native) and low oxygen prepared peptide (+16Da, +14Da, native). Insets: Fragment ion mass spectra for retention times indicated by the red dot.

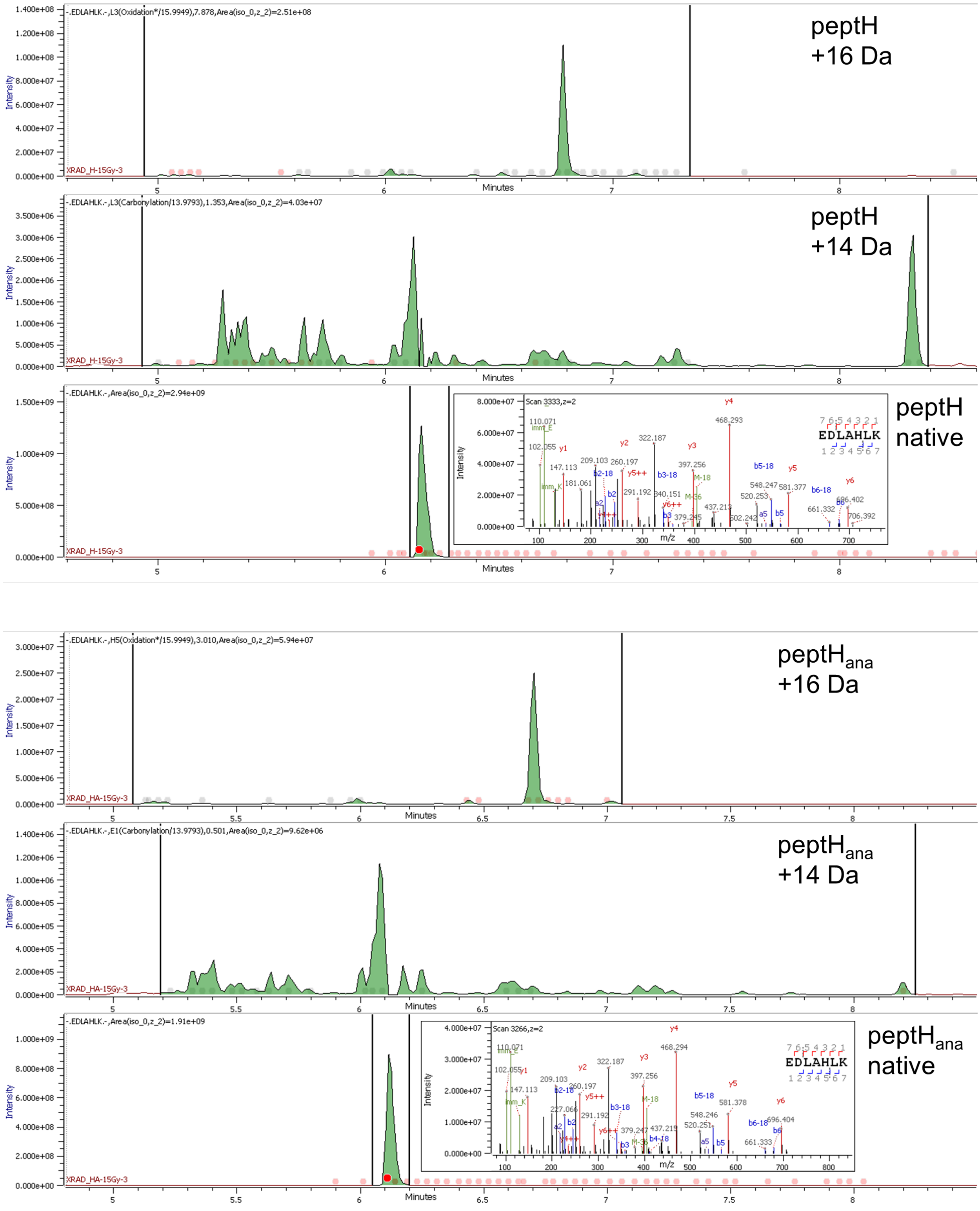

Figure S2: Extracted ion chromatograms (XICs) and representative MS/MS plots for peptH. From top to bottom: XICs for aerobically prepared peptide (+16Da, +14Da, native) and low oxygen prepared peptide (+16Da, +14Da, native). Insets: Fragment ion mass spectra for retention times indicated by the red dot.

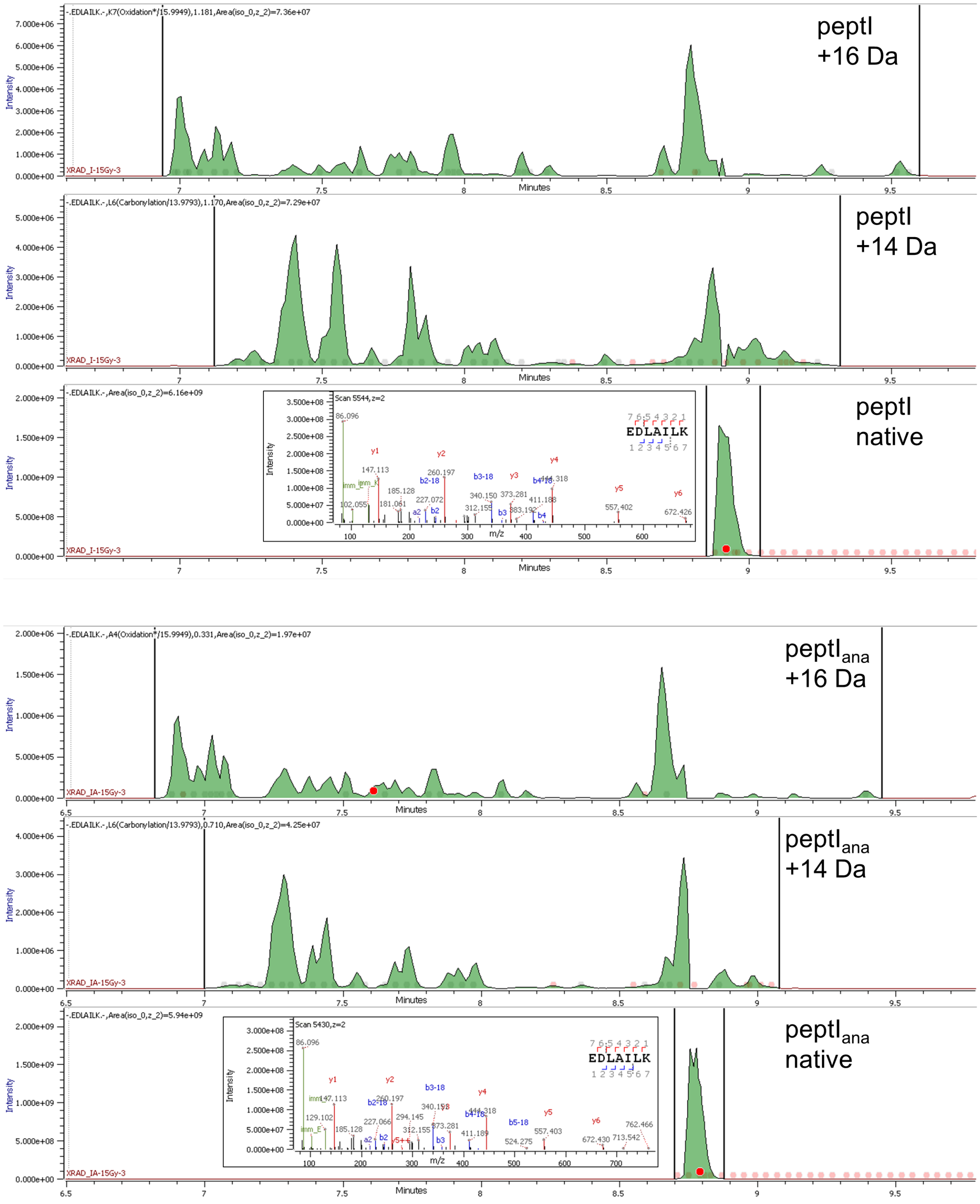

Figure S3: Extracted ion chromatograms (XICs) and representative MS/MS plots for peptI. From top to bottom: XICs for aerobically prepared peptide (+16Da, +14Da, native) and low oxygen prepared peptide (+16Da, +14Da, native). Insets: Fragment ion mass spectra for retention times indicated by the red dot.

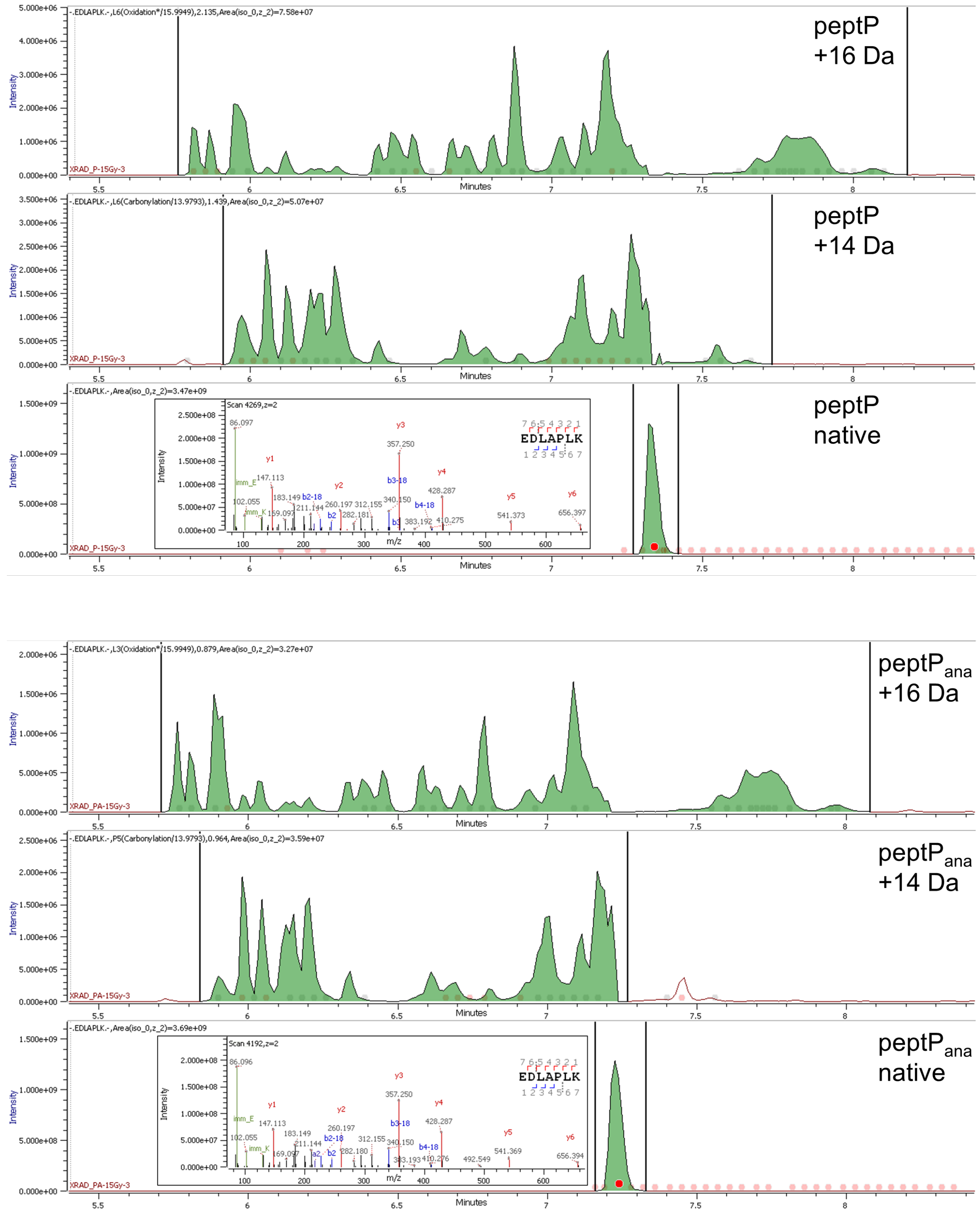

Figure S4: Extracted ion chromatograms (XICs) and representative MS/MS plots for peptP. From top to bottom: XICs for aerobically prepared peptide (+16Da, +14Da, native) and low oxygen prepared peptide (+16Da, +14Da, native). Insets: Fragment ion mass spectra for retention times indicated by the red dot.

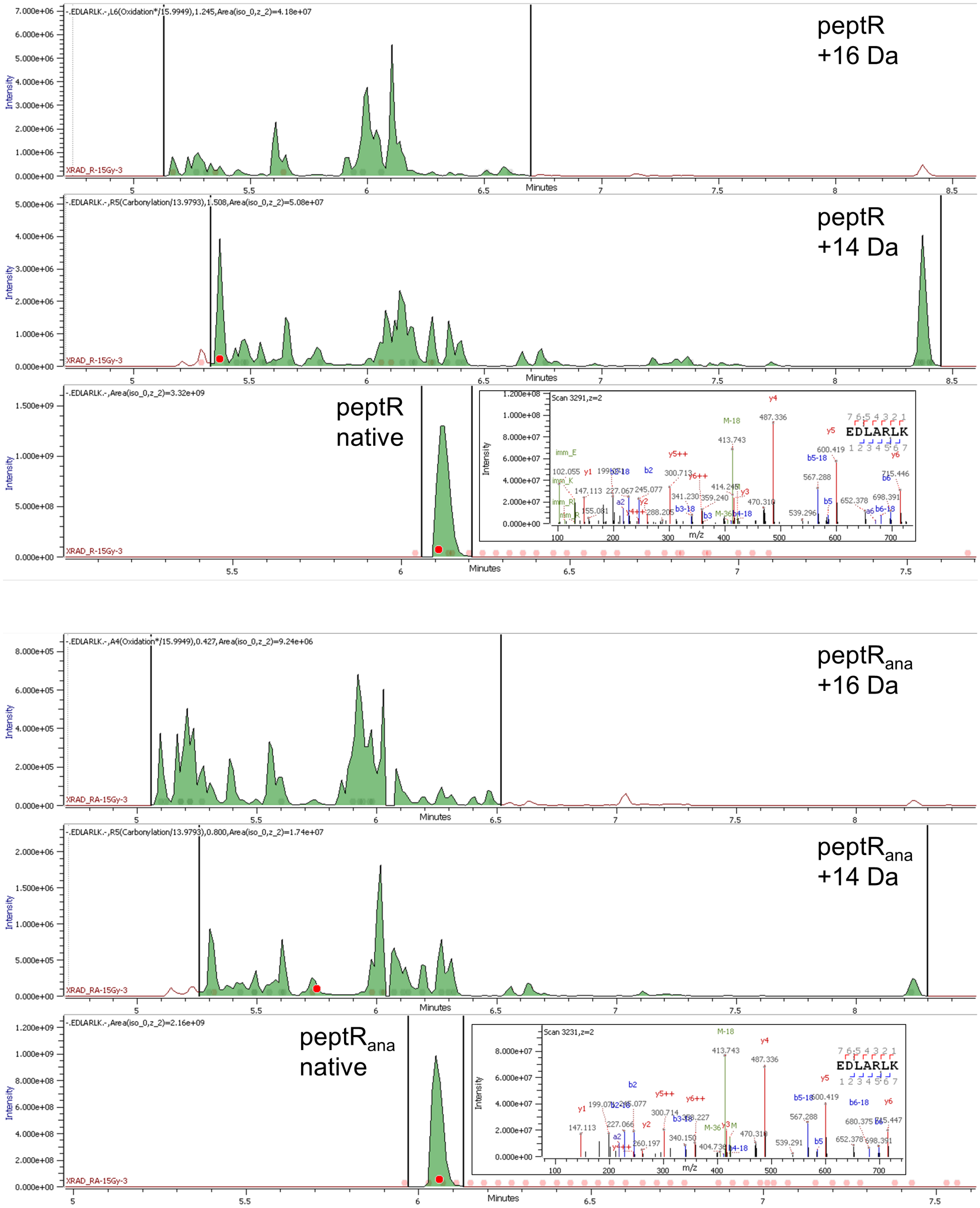

Figure S5: Extracted ion chromatograms (XICs) and representative MS/MS plots for peptR. From top to bottom: XICs for aerobically prepared peptide (+16Da, +14Da, native) and low oxygen prepared peptide (+16Da, +14Da, native). Insets: Fragment ion mass spectra for retention times indicated by the red dot.

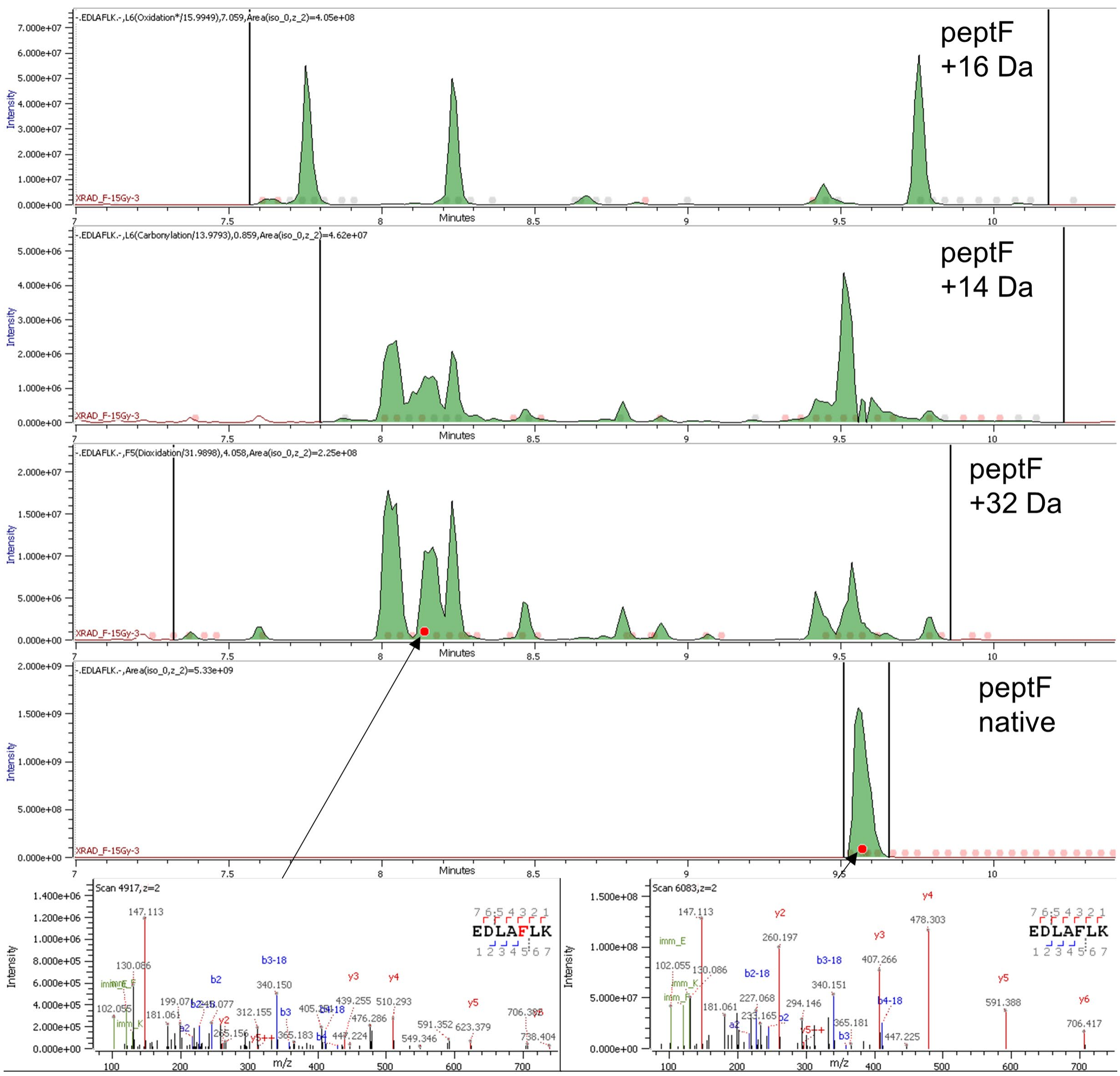

Figure S6: Extracted ion chromatograms (XICs) and representative MS/MS plots for aerobically prepared peptF. From top to bottom: +16Da, +14Da, +32Da and native XICs. Insets: Fragment ion mass spectra for retention times indicated by the red dot.

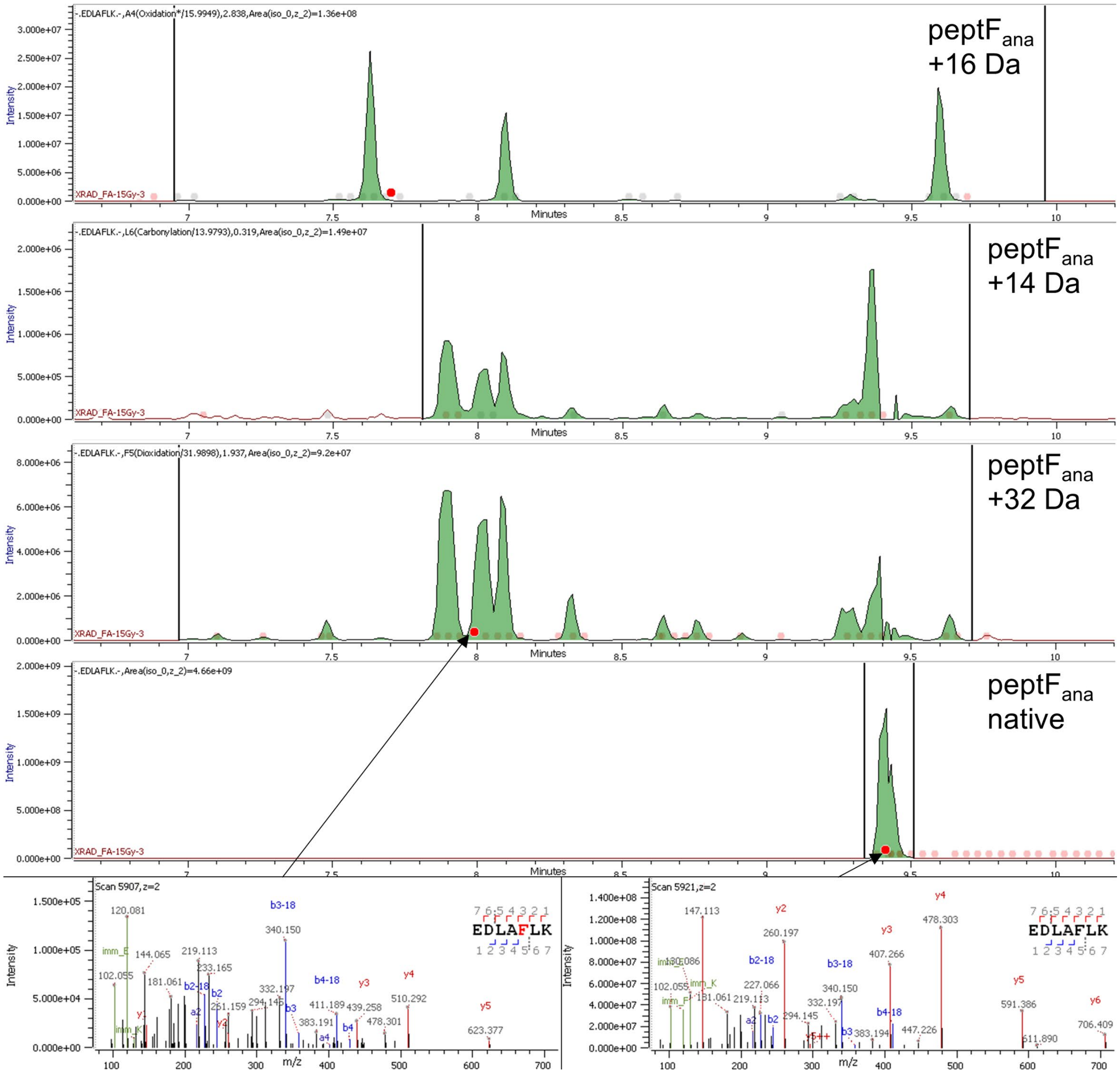

Figure S7: Extracted ion chromatograms (XICs) and representative MS/MS plots for low oxygen prepared peptF. From top to bottom: +16Da, +14Da, +32Da and native XICs. Insets: Fragment ion mass spectra for retention times indicated by the red dot.

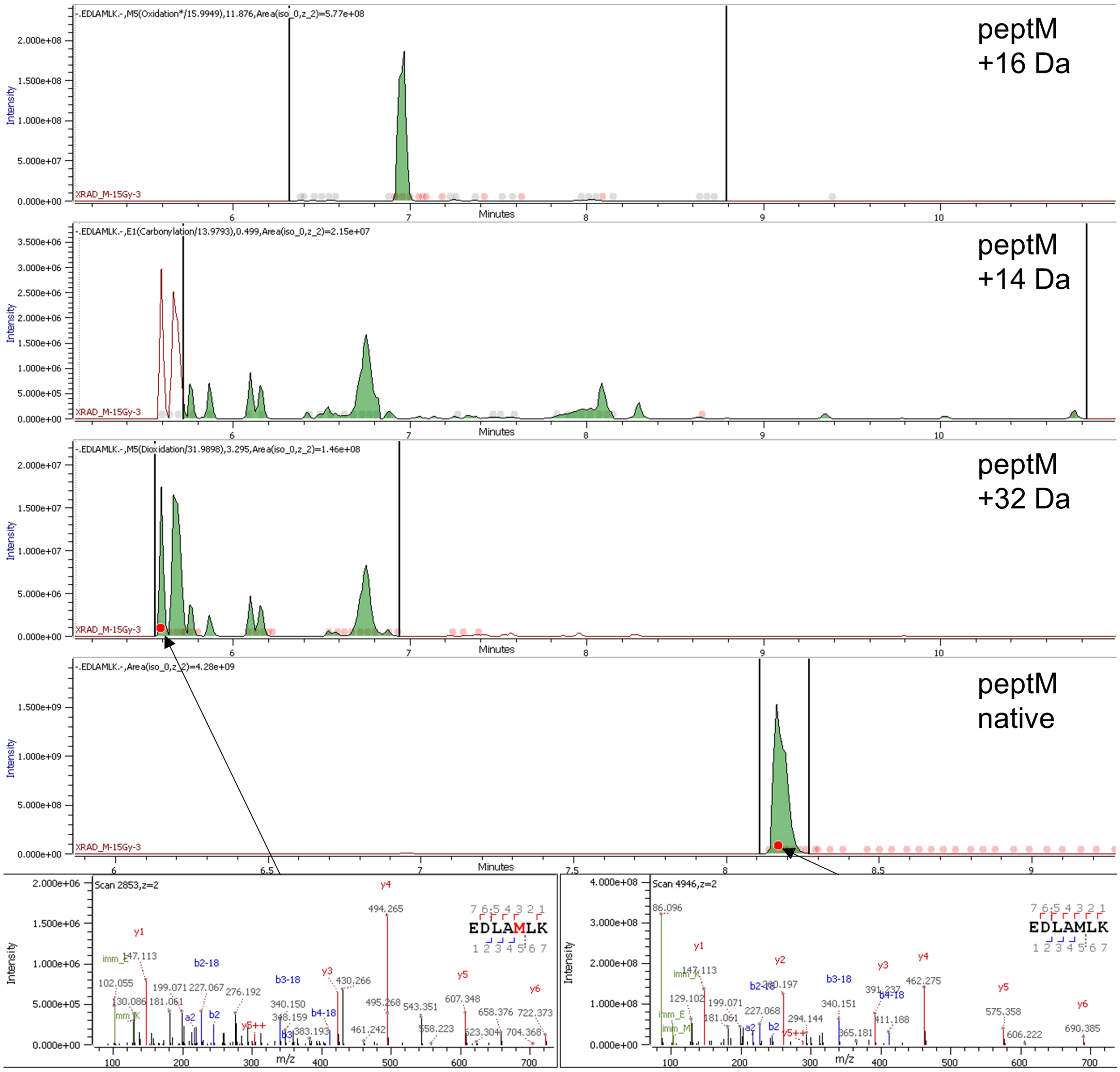

Figure S8: Extracted ion chromatograms (XICs) and representative MS/MS plots for aerobically prepared peptM. From top to bottom: +16Da, +14Da, +32Da and native XICs. Insets: Fragment ion mass spectra for retention times indicated by the red dot.

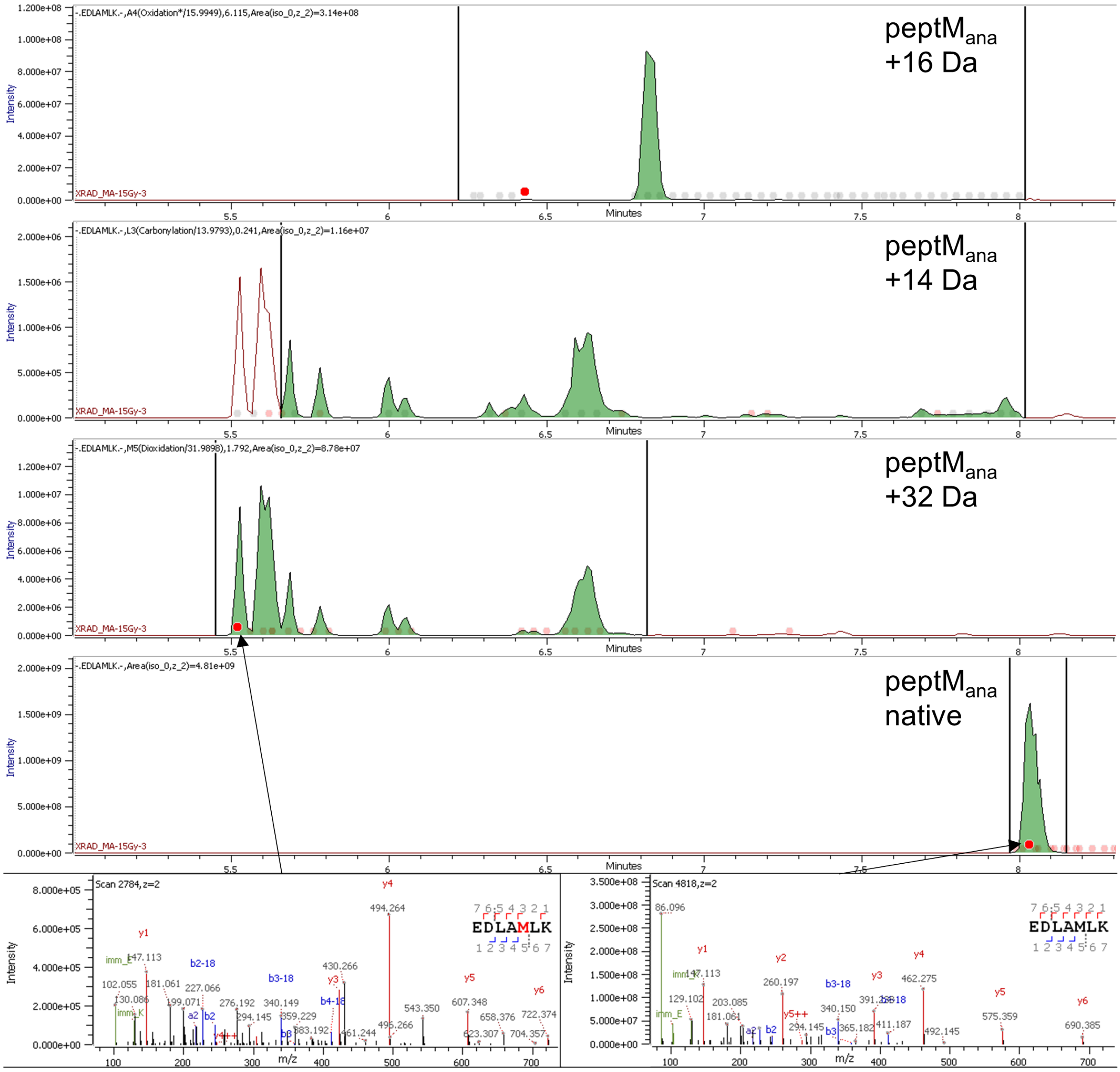

Figure S9: Extracted ion chromatograms (XICs) and representative MS/MS plots for low oxygen prepared peptM. From top to bottom: +16Da, +14Da, +32Da and native XICs. Insets: Fragment ion mass spectra for retention times indicated by the red dot.

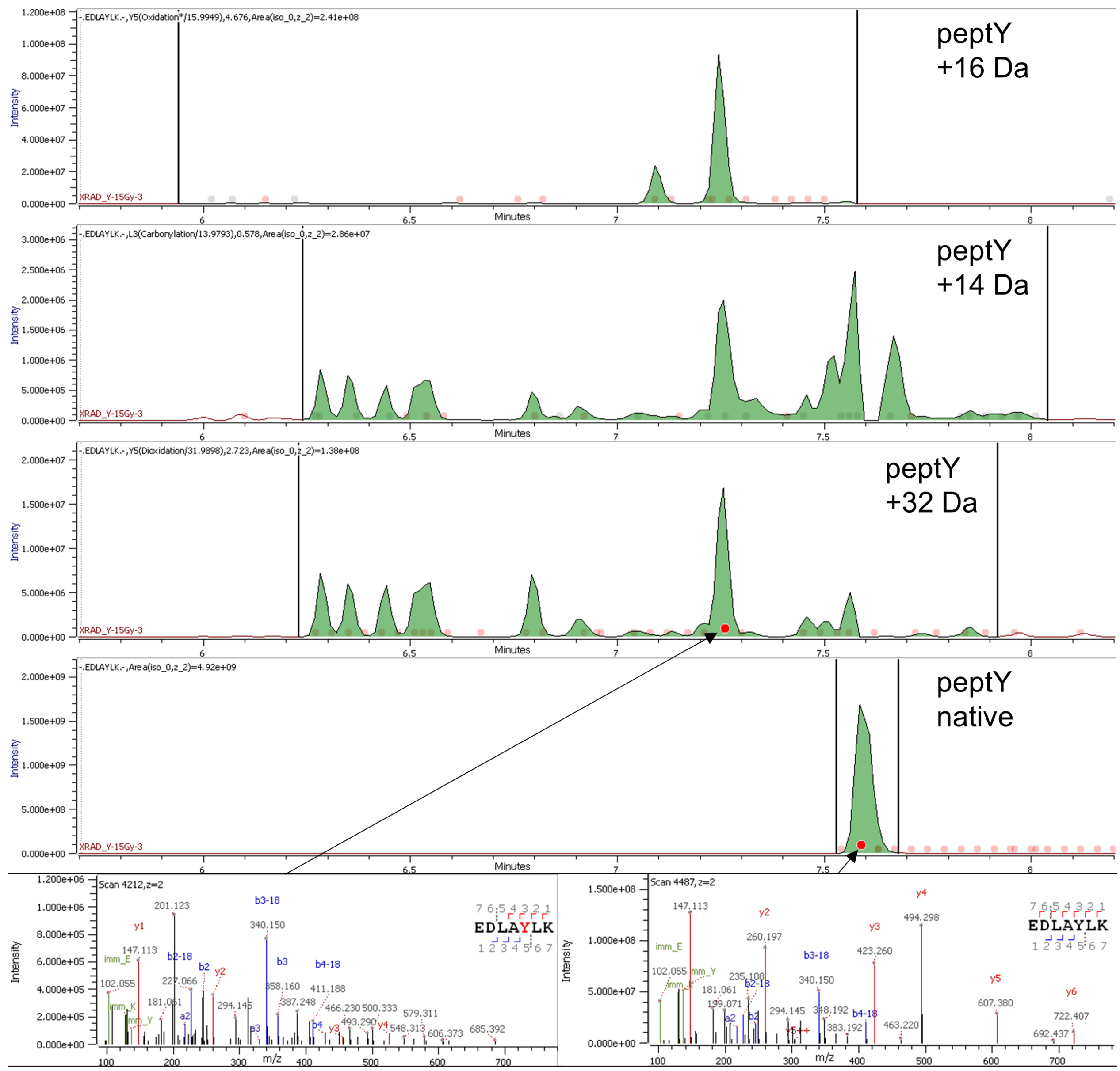

Figure S10: Extracted ion chromatograms (XICs) and representative MS/MS plots for aerobically prepared peptY. From top to bottom: +16Da, +14Da, +32Da and native XICs. Insets: Fragment ion mass spectra for retention times indicated by the red dot.

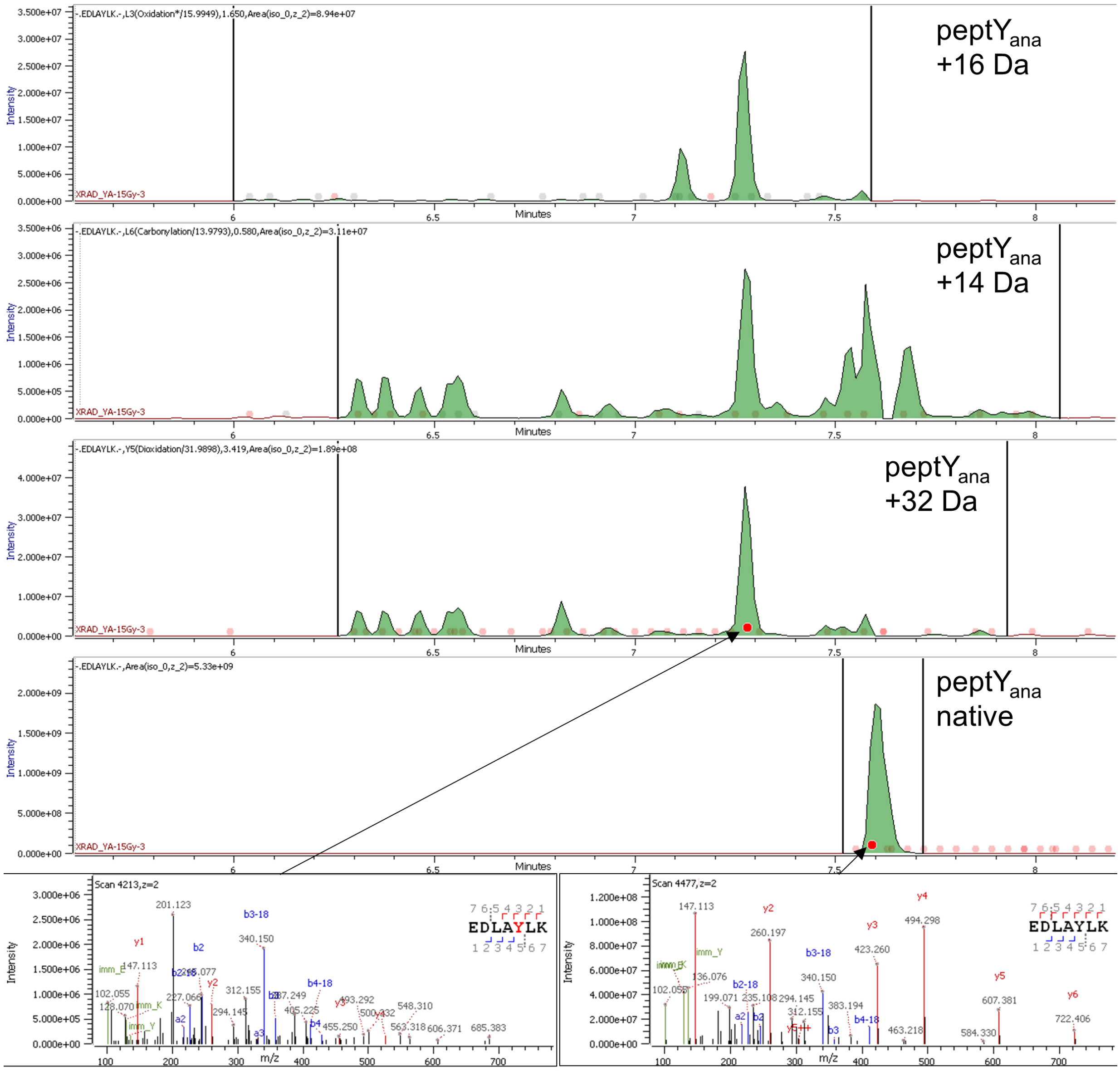

Figure S11: Extracted ion chromatograms (XICs) and representative MS/MS plots for low oxygen prepared peptY. From top to bottom: +16Da, +14Da, +32Da and native XICs. Insets: Fragment ion mass spectra for retention times indicated by the red dot.
